# Urbanization alters plastic responses in the common dandelion *Taraxacum officinale*

**DOI:** 10.1002/ece3.6176

**Published:** 2020-03-27

**Authors:** Matti Pisman, Dries Bonte, Eduardo de la Peña

**Affiliations:** ^1^ Terrestrial Ecology Unit (TEREC) Department of Biology Ghent University Gent Belgium; ^2^ Institute for Subtropical and Mediterranean Horticulture Finca Experimental La Mayora Spanish National Research Council (IHSM‐UMA‐CSIC) Malaga Spain

**Keywords:** biomass, evolved reaction norms, herbivory, seed morphology, *Taraxacum officinale*, urbanization

## Abstract

Urban environments expose species to contrasting selection pressures relative to rural areas due to altered microclimatic conditions, habitat fragmentation, and changes in species interactions. To improve our understanding on how urbanization impacts selection through biotic interactions, we assessed differences in plant defense and tolerance, dispersal, and flowering phenology of a common plant species (*Taraxacum officinale*) along an urbanization gradient and their reaction norms in response to a biotic stressor (i.e., herbivory). We raised plants from 45 lines collected along an urbanization gradient under common garden conditions and assessed the impact of herbivory on plant growth (i.e., aboveground biomass), dispersal capacity (i.e., seed morphology), and plant phenology (i.e., early seed production) by exposing half of our plants to two events of herbivory (i.e., grazing by locusts). Independent from their genetic background, all plants consistently increased their resistance to herbivores by which the second exposure to locusts resulted in lower levels of damage suffered. Herbivory had consistent effects on seed pappus length, with seeds showing a longer pappus (and, hence, increased dispersal capacities) regardless of urbanization level. Aboveground plant biomass was neither affected by urbanization nor herbivore presence. In contrast to consistent responses in plant defenses and pappus length, plant fitness did vary between lines. Urban lines had a reduced early seed production following herbivory while rural and suburban lines did not show any plastic response. Our results show that herbivory affects plant phenotypes but more importantly that differences in herbivory reaction norms exist between urban and rural populations.

## Graphical abstract

We assessed differences in plant traits in a common plant species (*Taraxacum officinale*) originating from different lines along an urbanization gradient and their reaction norms in response to a biotic stressor (i.e., herbivory) under common garden conditions. We found that differences in herbivory reaction norms exist between urban and rural populations, but they seem to be trait‐dependent.

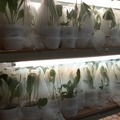

## 1 INTRODUCTION

One of the most prominent anthropogenic changes is the rapid expansion of urbanized areas, a type of ecosystem that vastly differs from natural ones in terms of environmental conditions and landscape structure. Urban environments are characterized by higher temperatures (urban heat island) and increased levels of light, sound, and chemical pollution. Furthermore, green spaces are rare, more enclosed by urban infrastructure, and frequently disturbed by human activity (Parris, 2016; Pickett et al., 2001). Urbanization subjects organisms to new selection pressures and is therefore anticipated to impose genetic divergence in species traits (Alberti, Marzluff, & Hunt, 2017; Johnson & Munshi‐South, 2017). As urban landscape composition and structure differs from pristine natural areas both at the local scale (e.g., local differences in patch quality vs. more homogenous green spaces (Cadenasso, Pickett, & Schwarz, 2007)) and at the landscape scale (e.g., increased fragmentation due to smaller patches and the concrete matrix (Cheptou, Carrue, Rouifed, & Cantarel, 2008; Dubois & Cheptou, 2017) such altered selective pressures can be expected at multiple spatial scales (Cheptou, Hargreaves, Bonte, & Jacquemyn, 2017). Qualitative changes in biotic interactions due to altered species composition or more quantitative changes in the functional responses are also anticipated to impose selection on traits related to species interactions (El‐Sabaawi, 2018; Moreira et al., 2019; Start, Bonner, Weis, & Gilbert, 2018). Aside from driving intraspecific trait shifts between populations along an urbanization gradient, biotic interactions can also affect phenotypic expression of traits through plasticity (Agrawal, Conner, Johnson, & Wallsgrove, 2002). The reaction norms underlying this plasticity may be subjected to selection as well (GxE interactions) (Via & Lande, 1985), thereby facilitating adaptation to urbanization.

Urbanization affects insect species richness and size (Merckx et al., 2018; Piano et al., 2017) and consequently herbivore pressure, but its direction and strength varies among species and locations (Raupp, Shrewsbury, & Herms, 2010). Previous work has found that plants are known to react to herbivory through the induction of defenses (Agrawal & Karban, 1999; Karban, 2011; Poelman, Broekgaarden, Loon, & Dicke, 2008), increased seed dispersal (Bonte et al., 2012; de la Pena & Bonte, 2014), and shifting flowering phenology (Agrawal, Strauss, & Stout, 1999; Rusman, Poelman, Nowrin, Polder, & Lucas‐Barbosa, 2019). The induction of defenses by spines or toxins, or the developmental of tolerance is energetically costly and is expected to trade‐off with vital traits as seed production and/or seed mass (Agrawal et al., 1999; Stearns, 1989). Escape strategies by changing growth or seed dispersal morphology (Bonte et al., 2012; Johnson et al., 2019; de la Pena & Bonte, 2014) can equally levy costs and lead to trade‐offs with other life‐history traits. These plastic responses can be expressed during ontogeny, or among generations through maternal effects, and may be subjected to selection as well (Saastamoinen et al., 2018). Given that herbivore pressure can differ along urban–rural gradients, it can be assumed that induction of defensive responses can shift following urbanization (e.g., Moreira et al. (2019)). Other plant life‐history traits have also been found to differ between urban and rural areas. Increased levels of fragmentation in urban areas may select for reduced dispersal capacities through for instance decreased investments in dispersal structures (i.e., wings, pappus length), as long‐distance dispersal in fragmented areas can have a higher cost due to increased mortality rates when seeds land in hostile landscape patches (Bonte et al., 2012; Cheptou et al., 2017; Jacquemyn, Meester, Jongejans, & Honnay, 2012). Such intraspecific differences in dispersal capacities following fragmentation in urban areas have been described (Cheptou et al., 2008; Riba et al., 2009), yet the strength of selection will depend on several factors (Cheptou et al., 2017). Differences in plant phenology between rural and urban areas have been observed under common garden conditions, yet drivers of these changes have not been identified so far (Yakub & Tiffin, 2017).

When both selection and plasticity act jointly, they do not always shape trait changes in the same direction. Gorton, Moeller David, and Tiffin (2018), for example, observed counter‐gradient variation between the genetic base and plastic responses driven by environmental conditions in plants in urban areas, suggesting opposite directions between adaptation and plastic responses to environmental variation. These results highlight the importance to assess how plasticity in response to stress and genetic selection in response to urbanization affect trait expression.

Here, we engaged in such a study using the common dandelion *Taraxacum officinale* as a model species. The species reproduces apomictic in Belgium, and all seeds are therefore clonal variants of the mother. We assessed (a) whether clonal variation exists in plant defense, dispersal capacity, and flowering phenology along an urbanization gradient and (b) whether or not reaction norms for these traits vary in relation to urbanization at different spatial scales.

We reared *T. officinale* collected along an urbanization gradient from seeds of single mothers in a common garden experiment to eliminate all direct environmental differences in trait expression. We used a hierarchical sample design with different levels of local urbanization nested within different levels of urbanization at the landscape scale, to account for effects of urbanization at both a local and landscape scale. This design allowed us to assess the scale at which urbanization would influence our investigated life‐history traits and whether or not responses differed between different spatial scales. We exposed part of the plants to insect herbivory, to investigate whether this exposure causes plasticity in a selection of relevant life‐history traits along an urbanization gradient, and if so, whether the reaction norms differ in relation to urbanization.

## 2 MATERIAL AND METHODS

### 2.1 Study design

Our study was conducted in the northern part of Belgium, one of the most urbanized regions in Europe. We used a hierarchically nested sampling design where local‐scale levels of urbanization (subplots, 200 m × 200 m) were repeatedly sampled across different landscape‐scale levels of urbanization (plots, 3,000 m × 3,000 m). The level of urbanization for each plot and subplot was determined based on the percentage of the total area covered with buildings (Rural: 0%–3% buildings and at least 20% biologically valuable area, suburban: 5%–10% buildings, and urban: >15% buildings). We selected three plots located in the province of East Flanders for each level of urbanization, for a total of nine plots. Within each plot, we selected two rural subplots, one suburban subplot, and two urban subplots, yielding a total of 45 locations. Our plots were a subset of the experimental setup used in earlier studies which found differences in insect communities along an urbanization gradient (Merckx et al., 2018; Piano et al., 2017). A full overview of the sampled locations is given in Table S1.

### 2.2 Model organisms

#### 2.2.1 *Taraxacum officinale* as a model organism

The common dandelion or *Taraxacum officinale* is a herbaceous perennial plant belonging to the family of the Asteraceae. The species is native to Europe and Asia but can be found worldwide in temperate climates (Berg, 2007). *Taraxacum officinale* is a ruderal species, which can be commonly found in highly urbanized areas. It is characterized by a large taproot, a flower head consisting of multiple small yellow flowers and a basal rosette of leaves. Its seeds are wind dispersed, and individuals growing in northern Europe are apomictic, meaning their seeds are formed without fertilization, resulting in a clonal reproduction through seeds that are genetically identical to the mother (Weeda, 1987). Seeds are wind dispersed, being the majority of seeds dispersed within 10 m of the mother plant, yet long‐distance dispersal (LDD) events can exceed 100 m for a small percentage of seeds (Tackenberg, Poschlod, & Kahmen, 2003b). We therefore expect most seeds to disperse within the close vicinity of the mother plants, but events of LDD across the border of our subplots cannot be excluded completely. By using *T. officinale* as a model organism, we ensured that plants grown in the laboratory had the same genotype as the mother plants in the field and represent the same clonal line.

#### 2.2.2 *Schistocerca gregaria* as an aboveground herbivore

The desert locust *Schistocerca gregaria* (Orthoptera) is a generalist herbivore which feeds on a large array of plants and plant parts, causing mechanical damage through chewing. This species was selected as earlier research already showed that this locust is a suitable model species for herbivory experiments with dandelion (de la Pena & Bonte, 2014). As it is a non‐native herbivore to our study region, possible influences of coevolution between *T. officinale* and the insect are avoided. Individuals used in the experiment were bought from a mass‐rearing program, and all had the same age, to minimize the effect of individual variation among the locusts. The use of these individuals avoids potential effects due to differences in the timing of the two exposures, as individuals were directly transferred from the mass‐rearing conditions to the common garden conditions that were identical between the two exposure periods.

### 2.3 Seed collection

Seeds were collected in September 2014 in all of our 45 selected locations. At each location, we sampled multiple seed heads from single mother plants. Seeds from each location were considered as distinct clones and are from here on referred to as lines. Seeds were put in labeled paper envelops and kept at room temperature until the start of the experiments.

### 2.4 Common garden experiment

#### 2.4.1 Raising the experimental plants

At the end of September 2014, seeds from the 45 lines were transferred to plastic containers filled with wet cotton at the bottom. After 2 weeks, seedlings from 44 lines were transplanted to 1‐L pots filled with potting soil. Seven pots were prepared per sample site, each initially containing five seedlings. The pots were then placed in a breeding chamber at 23°C under a 16 hr light/8 hr dark photoperiod regime to allow the seedlings to grow for another 2 weeks. At the end of October 2014, the largest seedling in each pot was selected while the others were removed, leaving seven genetically identical plants per line to be used in the experiment, for a total of 308 plants. Each seedling was then randomly assigned to either the control group (three replicas per line) or the herbivory treatment group (four replicas per line). Plants from both the control and herbivory treatment group were put randomly in a growth chamber under the same conditions as described above. Plants were watered once every week until soil saturation and fertilized two times (at the end of November 2014 and December 2014, respectively) with 1 ml central park bio fertilizer (NPK 7‐3‐6). The experiment lasted from October 2014 till June 2015. At the end of the experiment, 285 out of the initial 308 plants remained for data collection.

#### 2.4.2 Experimental treatment with locusts

Aboveground grazing by *S. gregaria* took place in December 2014 and February 2015. Plants from both the control and herbivory treatment were enclosed with a net, and one locust was put on each plant from the herbivory treatment, while the nets around the control plants remained empty. Locusts were allowed to graze ad libitum for 2 weeks, after which they were removed. Between the two locust grazing periods, the nets were removed to prevent interference with plant growth and recovery.

### 2.5 Trait quantification

#### 2.5.1 Quantifying herbivore damage as a proxy for defensive responses

Upon removal of the locusts, we quantified the severity of the damage to the plants in the herbivory treatment group by assigning each plant to 1 of 4 predefined categories (s0 = no damage to any of the leaves, s1 = minor damage to at least 1 leaf, s2 = severe damage to at least 1 leaf, s3 = complete defoliation of the plant). We also counted the number of leaves showing signs of damage (regardless of how severe) and the amount of leaves that remained untouched, to calculate the percentage of leaves that suffered grazing by the locusts.

#### 2.5.2 Dry aboveground biomass weight

At the end of the experiment, the leaves of all the plants were cut from the root and stored into envelops. These were then placed in an oven to dry, after which the remaining dry biomass was weighted using an Ohaus Galaxy G 110 Digital Balance to measure the aboveground production of the plants.

#### 2.5.3 Early reproductive output

We quantified the total reproductive output of each plant over a period of 15 weeks and assessed whether or not different seed packaging strategies (size–number trade‐offs) existed among our populations. Collection of seed heads started as soon as the first head was formed among all the flowers. Seed heads were checked daily to ensure new heads were collected before losing any seeds. Collected seed heads were kept in separate labeled paper envelops at room temperature. After 15 weeks, each seed head was weighed and the number of seeds counted. For each plant, we determined the total seed weight, the total number of seed heads, and the average number of seeds per seed head. We assessed the effect of urbanization and herbivore treatment on all measured traits. Plants that did not form any flowers during the 15‐week period were excluded from the analyses.

#### 2.5.4 Seed morphology

For low growing plants such as *T. officinale*, altering the seed terminal velocity is one of the most effective strategies to change the dispersal capacity (Tackenberg, Poschlod, & Bonn, 2003a), as this determines the chance to get uplifted by wind. Changing seed terminal velocity can be achieved by modifying the ratio of the pappus area and seed weight. Consequently, we measured pappus length, total propagule length, and seed size to assess potential differences in seed morphology related to dispersal distance. For each plant that flowered in the common garden, we selected five seeds at random to investigate seed morphology. All seeds were scanned using a Canoscan Lide 100 scanner, and images were analyzed using ImageJ. Total propagule length was calculated as the sum of seed length and beak length. Seed size was calculated using the length and width of the seed assuming that the seeds are cylindrical in shape.

### 2.6 Statistical analyses

Results were analyzed using either linear mixed models (LMM) or generalized linear mixed models (GLMM), depending on the investigated trait (LMM for normal error structure, GLMM for binomial, multinomial, or Poisson error structure). We used a full factorial model which included the fixed factors “Treatment” (herbivores absent or present), “Plot” (landscape level urbanization: urban, suburban, or rural), and “Subplot” (local level urbanization: urban, suburban, or rural), as well as their interactions. For analyzing the data on herbivore damage, we also included the fixed factor “Timing” to assess the difference between the first and second exposure to locusts. We also included the line of origin as random effect to control for common clonal background of the plants.

All analyses were performed using the SAS (9.4) software procedures “PROC MIXED” (LMM) and “PROC GLIMMIX” (GLMM). The effective degrees of freedom were estimated by the Satterthwaite's approximation (Verbeke & Molenberghs, 2009). Type III tests of Fixed Effects were performed for backwards model selection, starting from the full factorial model. Nonsignificant parameters were removed until the final model only contained factors and interactions that were significant (*p* < .05). When a trait was significantly affected by a certain factor, we used a Tukey's post hoc HSD test to assess the differences between the respective groups.

## 3 RESULTS

### 3.1 Herbivore damage

**Figure 1:**
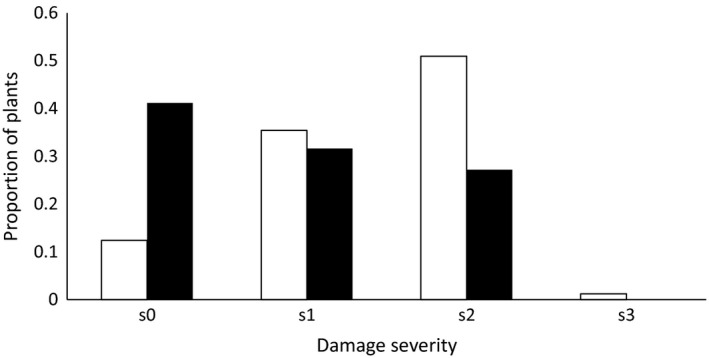
Quantification of herbivore damage. Proportion of the experimental plants exposed to herbivores assigned to each category (s0: no damage to any leaf, s1: minor damage to at least one leaf, s2: severe damage to at least one leaf, s3: complete defoliation of the plant) after the first (white bars, total plants: 161) and second (black bars, total plants: 158) exposure to *Schistocerca gregaria*

**Figure 2:**
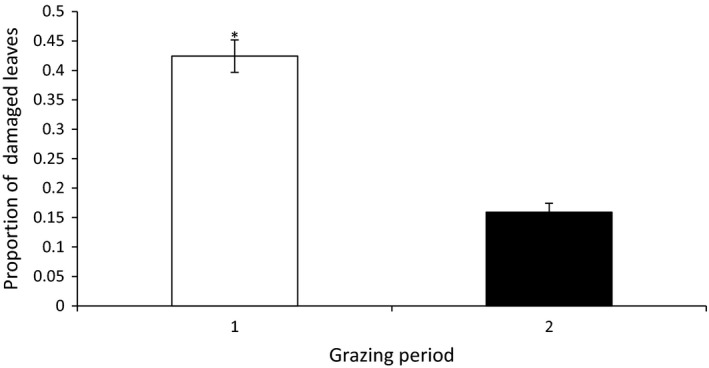
Proportion of leaves per plant damaged by locusts presented as least square means and standard error. White bar indicates damage suffered during the first grazing period, and black bar indicates damage suffered during the second grazing period. Bars marked with an asterisk differ significantly according to Tukey's post hoc test (*p* ≤ .05). Average number of leaves per plant right before the first and second exposure was 8.44 ± 1.71 and 11.41 ± 3.97 (Mean ± *SD*), respectively

Urbanization did not affect herbivore damage (severity) at either the landscape (*F*_(2, 313)_ = 0.22, *p* = .79) or local scale (*F*_(2, 315)_ = 1.88, *p* = .15). Herbivory timing did have an effect (*F*_(1, 317)_ = 34.21, *p* < .0001), and in the second exposure, herbivore damage was significantly lower than in the first one (Figure 1). The same pattern was found for the analysis of the proportion of damaged leaves: urbanization at the landscape (*F*_(2, 9.378)_ = 0.08, *p* = .92) or local (*F*_(2, 136.8)_ = 0.63, *p* = .54) scale did not affect the percentage of leaves that were grazed upon, so urbanization had no effect. Again, timing (*F*_(1, 313)_ = 307.15, *p* < .0001) did have a significant effect, as the percentage of leaves damaged in the second exposure was significantly lower than in the first one (Figure 2)*.* None of the interactions between the three factors were significant (*p* > .05) (Table S2).

### 3.2 Dry aboveground biomass weight

**Figure 3:**
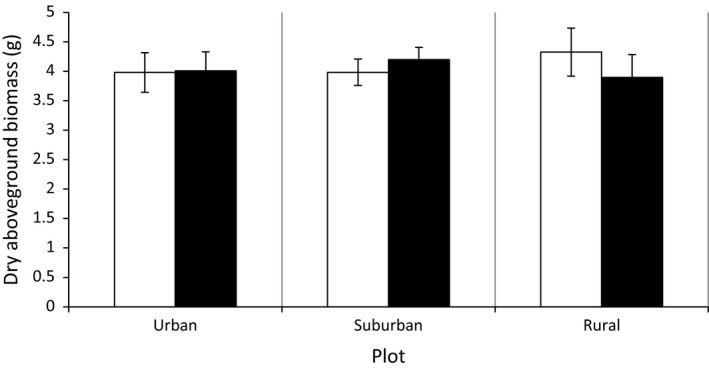
Least square means and standard error for the dry aboveground biomass per urbanization level at the landscape scale. White bars indicate values for the control group; black bars indicate values for the herbivore treatment group. White bars marked with an asterisk differ significantly from the black bars within the same urbanization level according to Tukey's post hoc test (*p* ≤ .05)

Dry aboveground biomass production was not significantly affected by urbanization on the landscape (*F*_(2, 17.4)_ = 0.06, *p* = .95) nor the local scale (*F*_(2, 20.5)_ = 1.01, *p* = .38). We could neither detect a statistical significant effect of herbivory (*F*_(1, 257)_ = 0.04, *p* = .84) or any interaction between the factors (*p* > .05) (Figure 3, Table S3).

### 3.3 Plant reproduction

**Figure 4:**
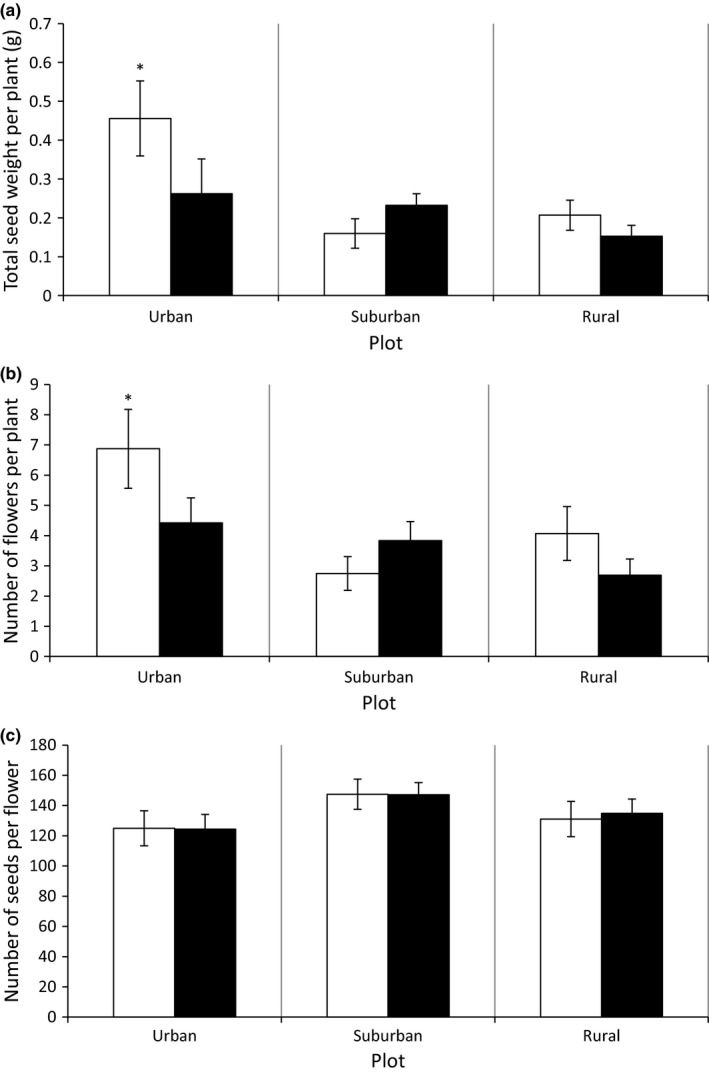
Least square means and standard error of (a) Total seed weight per plant (g), (b) Number of flowers per plant, and (c) Number of seeds per flower per urbanization level at the landscape scale. White bars indicate values for the control group; black bars indicate values for the herbivore treatment group. White bars marked with an asterisk differ significantly from the black bars within the same urbanization level according to Tukey's post hoc test (*p* ≤ .05)

Over the course of 15 weeks, 118 of the 292 plants (40.41%) flowered (i.e., yielded at least one flower with seeds). Flowering of plants occurred randomly and was not influenced by the urbanization level or the treatment (Plot: *F*_(2, 5.343)_ = 0.39, *p* = .6934, Subplot: *F*_(2, 10.68)_ = 2.34, *p* = .1440, Treatment: *F*_(1, 290)_ = 3.01, *p* = .0837). Looking at the total seed weight per plant, we found a significant interaction between herbivory treatment and urbanization at the landscape scale (*F*_(2, 98.9)_ = 6.38, *p* = .0025). The same significant interaction was found for the number of seed heads per plant (*F*_(2, 112)_ = 6.77, *p* = .0017). A full overview of the two analyses is given in the supporting information (Table S4). Total seed weight and the number of seed heads per plant were both higher in the control group compared to the herbivory group in urban areas, while populations originating from rural or suburban areas did not show this pattern (Figure 4a,b; see also Table S5). The number of seeds per seed head did not differ along the urbanization gradient nor was it affected by herbivore grazing (Figure 4c, Table S4).

### 3.4 Seed morphology

Seed size and total propagule length were both unaffected by the herbivore treatment (Table 1). Plants from the herbivory treatment developed longer pappus hairs compared to the control plants (Treatment: 0.607 ± 0.01 cm, Control: 0.586 ± 0.01 cm (LSMeans ± *SE*)), independent of the urbanization level (Table 1).

**Table 1:** Results of the Type III tests of Fixed Effects used for backwards model selection of the linear mixed models, testing the effect of plot, subplot, and treatment on (1) pappus length, (2) total propagule length, and (3) seed size

	Pappus length LMM		Propagule length LMM		Seed size LMM	
	*F*	*p*	*F*	*p*	*F*	*p*
Plot	*F*_(2, 12.8)_ = 1.13	.3543	*F*_(2, 566)_ = 1.12	.3264	*F*_(2, 4.23)_ = 0.48	.6503
Subplot	*F*_(2, 12.9)_ = 0.06	.9414	*F*_(2, 108)_ = 0.34	.7104	*F*_(2, 87.1)_ = 0.98	.3784
Treatment	*F*_(1, 95.8)_ = 4.55	**.0354**	*F*_(1, 568)_ = 1.57	.2106	*F*_(1, 106)_ = 0.39	.5335
Plot*Subplot	*F*_(4, 10.3)_ = 0.58	.6815	*F*_(4, 100)_ = 0.43	.7883	*F*_(4, 96.6)_ = 0.29	.8846
Plot*Treatment	*F*_(2, 93)_ = 0.48	.6196	*F*_(2, 562)_ = 1.57	.2102	*F*_(2, 104)_ = 1.56	.2140
Subplot*Treatment	*F*_(2, 94.2)_ = 0.26	.7688	*F*_(2, 104)_ = 0.58	.5623	*F*_(2, 102)_ = 1.02	.3632
Plot*Subplot*Treatment	*F*_(3, 88.6)_ = 0.86	.4635	*F*_(3, 97)_ = 0.79	.5026	*F*_(3, 94.4)_ = 0.01	.9986

Values printed in bold indicate factors with a significant effect at the .05 significance level.

## 4 DISCUSSION

We experimentally demonstrated a significant interaction of urbanization and insect herbivory on early seed production in *T. officinale*. This interaction indicates that differences in early seed production between the control and herbivory treatments were different for plants originating from different levels of urbanization, hence demonstrating clonal and genetic variation in plasticity in response to herbivore exposure between rural and urban populations. In contrast, herbivore exposure increased dispersal capacity (i.e., longer pappus) and herbivore resistance (i.e., reduced damage during a second exposure) in all lines equally, while total dry aboveground biomass was unaffected by both herbivore exposure or urbanization level. All differences observed along an urbanization gradient were found at the landscape‐scale level. Despite the fact that the majority of seeds in *T. officinale* are expected to disperse close to the mother plant, we could not completely exclude the possibility of LDD events between subplots given their spatial scale (200 m × 200 m areas). This could potentially facilitate gene flow between subplots and reduce genetic differentiation. As such, our local urbanization scale might be considered as an area in which dispersal between subplots is frequent enough to prevent differentiation in traits (Richardson, Urban, Bolnick, & Skelly, 2014). A second possibility is that drivers at the local scale are not prominent enough to drive genetic differentiation. Regardless of the underlying mechanism, we found no differences at the local scale, and from here on in the discussion, all comparisons between plants from different urbanization levels will refer to differences at the landscape scale.

We assessed the impact of herbivory by measuring the damage suffered during two separate exposures to herbivorous locusts and found a significant difference in the damage suffered by plants between the two herbivore exposures. For all lines, plants suffered significantly less damage during the second exposure, indicating a common underlying mechanism of induced resistance. We found no differences in damage severity among plants from different populations along an urbanization gradient, which implies that investment in defensive traits did not differ between different levels of urbanization. Aboveground plant biomass did also not differ significantly between populations along an urbanization gradient. As such, no differences in plastic responses were observed for plant defense and tolerance‐related traits between lines. As other plants have shown shifts in trait expression following herbivore exposure along an urbanization gradient (Moreira et al., 2019), this result goes against our expectations. However, we should note that we exposed our plants only to a generalist aboveground herbivore species, which induces damage through chewing. Other herbivores such as seed predators (Honek & Martinkova, 2005) and root predators (Huber et al., 2016) are known for *T. officinale* and can also induce defensive responses (de la Pena & Bonte, 2014). Therefore, it is possible that evolutionary trade‐offs between constitutive and induced responses against different types of herbivores which were not tested here could explain variation among studies and systems (Kempel, Schädler, Chrobock, Fischer, & Kleunen, 2011).

Lines from urban areas showed an increased early reproductive output (i.e., higher total seed mass) in absence of herbivory relative to those exposed to herbivores. The same pattern was found for the total number of seed heads produced, while the number of seeds per seed head and seed size was unaffected by either the herbivore treatment or urbanization level. This indicates that differences in total seed weight production were solely caused by the difference in number of seed heads that were formed. As we measured seed production during a 15‐week period in the early stage of reproduction, which is only a part of the total reproductive window for this species, these results imply that urban individuals of *T. officinale* allocate more resources to reproductive output in the early stage of reproduction in the absence of insect herbivores. However, no such patterns were found for dandelions originating from rural and suburban environments. Rural and suburban lines showed no difference in early reproductive output between plants exposed to locusts and the control group, indicating an absence of phenotypic plasticity in response to herbivore exposure. These results imply that the shape of reaction norms driven by biotic interactions can shift in response to urbanization. Shifts in reaction norms driven by abiotic conditions following urbanization have been described for fungi (McLean, Angilletta, & Williams, 2005), yet our study is the first to observe such patterns in plants under controlled conditions.

Dispersal‐related seed traits were significantly affected by the exposure to locusts. Seeds from plants exposed to locusts had a longer pappus length compared to those from the control group. Since seed size and propagule length did not differ between the treatment and control group, this would result in an increased pappus area relative to the seed size. This shift is expected to decrease the seed's terminal velocity and therefore increases the potential dispersal distance (Matlack, 1987). However, it remains hard to assess how big the impact on dispersal distance of these changes is. Due to the relatively small changes, the average dispersal distance will probably not be altered dramatically. However, as shown by Tackenberg, Poschlod, and Kahmen (2003), long‐distance dispersal (LDD) in *T. officinale* highly depends on strong updrafts. When these rare events that facilitate LDD occur, a decreased terminal velocity caused by a larger pappus area: seed weight ratio may further facilitate LDD and increase the chance for successful LDD. These findings are in line with the results of de la Pena and Bonte (2014) and confirm in a second set of experiments that a plastic response to increase dispersal distance in response to herbivory is present in *T. officinale*. As already shown in many other systems, stressful conditions experienced by the mother plant can result in an increased dispersive capacity of the seeds as a way to maximize fitness. Seeds that germinate further away from the mother plant are less prone to get predated by the herbivores experienced by the mother plant, increasing their chances to survive (Bonte et al., 2012; Nathan & Casagrandi, 2004). Similar to the defense and tolerance traits, this plastic response was also conserved across the urbanization gradient.

While we did not address experimentally the exact mechanisms through which urbanization has selected for shifts in the investigated traits, our results clearly show that changes in reaction norms exist along an urbanization gradient in the presence–absence of large herbivorous insects, yet responses seem to vary between plant traits. Plants from urban areas invested more in early flowering in the absence of herbivores, which might indicate a cost from herbivore damage. One potential explanation is a trade‐off with plant growth, where resources had to be allocated to restore the lost biomass, as aboveground biomass did not vary between damaged and undamaged plants (Agrawal et al., 1999). Regardless of the underlying mechanism, this plastic response in early seed production was absent in semi‐urban and rural populations. As *T. officinale* is apomictic in Belgium, we would expect that the observed changes either originated from the standing genetic variation in populations or through changes in DNA methylation affecting trait expression, as stress‐induced methylation in *T. officinale* can be triggered and passed on to the offspring, allowing for rapid changes in plant trait expression despite offspring being genetically identical to the mother plant (Verhoeven, Jansen, Dijk, and Biere (2010)).

In summary, our common garden study shows that variation of reaction norms driven by herbivore damage exists along an urbanization gradient in *T. officinale*. However, differences in plasticity were trait dependant as some plastic responses were conserved along the sampled urbanization gradient.

## CONFLICT OF INTEREST

The authors declare no conflict of interest.

## AUTHOR CONTRIBUTIONS

MP: experiments; data analysis; and writing – initial draft. DB and EDLP: experiment design; data analysis; and later versions of the draft. All authors contributed to the revision of the final draft and gave approval for publication.

## Supporting information

Table S1‐S5

## Data Availability

The data shown in this manuscript are deposited in the open data bank “DRYAD digital repository” (https://doi.org/10.5061/dryad.f1vhhmgt2).
