# Supplementary material for: Urbanization alters plastic responses in the common dandelion Taraxacum officinale
Source: Ecol Evol. 2020 Mar 27;10(9):4082–90. doi: 10.1002/ece3.6176 (PMC7244812; doi:10.1002/ece3.6176)
Supplement: Supplementary file 1 — Table S1‐S5 [file ECE3-10-4082-s001.docx]

**Supporting information**

| **Location** | **Landscape urbanization** | **Local urbanization** | **Latitude** | **Longitude** |
| --- | --- | --- | --- | --- |
| Aalst | Urban | Urban | 50°56'52.94"N | 4° 1'56.60"E |
| Aalst | Urban | Urban | 50°56'59.13"N | 4° 1'47.23"E |
| Aalst | Urban | Suburban | 50°57'53.33"N | 4° 2'19.91"E |
| Aalst | Urban | Rural | 50°57'32.55"N | 4° 2'16.56"E |
| Aalst | Urban | Rural | 50°57'9.81"N | 4° 1'1.12"E |
| Gent | Urban | Urban | 51° 3'6.69"N | 3°42'35.31"E |
| Gent | Urban | Urban | 51° 2'34.65"N | 3°42'1.10"E |
| Gent | Urban | Suburban | 51° 3'41.93"N | 3°44'10.94"E |
| Gent | Urban | Rural | 51° 2'50.45"N | 3°42'7.86"E |
| Gent | Urban | Rural | 51° 2'57.82"N | 3°41'48.53"E |
| Oudenaarde | Urban | Urban | 50°52'7.14"N | 3°36'20.67"E |
| Oudenaarde | Urban | Urban | 50°52'11.35"N | 3°36'43.68"E |
| Oudenaarde | Urban | Suburban | 50°51'46.22"N | 3°36'29.14"E |
| Oudenaarde | Urban | Rural | 50°52'0.99"N | 3°36'32.88"E |
| Oudenaarde | Urban | Rural | 50°52'1.24"N | 3°37'31.34"E |
| Beervelde | Suburban | Urban | 51° 3'24.24"N | 3°50'13.96"E |
| Beervelde | Suburban | Urban | 51° 4'30.48"N | 3°50'35.87"E |
| Beervelde | Suburban | Suburban | 51° 4'4.07"N | 3°50'24.75"E |
| Beervelde | Suburban | Suburban | 51° 3'22.94"N | 3°50'5.05"E |
| Beervelde | Suburban | Rural | 51° 4'3.52"N | 3°51'17.30"E |
| Beervelde | Suburban | Rural | 51° 4'13.41"N | 3°50'25.31"E |
| Bellem | Suburban | Urban | 51° 4'57.42"N | 3°26'41.05"E |
| Bellem | Suburban | Urban | 51° 5'5.85"N | 3°27'23.10"E |
| Bellem | Suburban | Urban | 51° 5'5.72"N | 3°27'26.29"E |
| Bellem | Suburban | Rural | 51° 4'35.43"N | 3°27'5.56"E |
| Bellem | Suburban | Rural | 51° 5'46.99"N | 3°27'41.80"E |
| Hillegem | Suburban | Urban | 50°53'12.26"N | 3°50'1.47"E |
| Hillegem | Suburban | Urban | 50°54'8.29"N | 3°50'13.41"E |
| Hillegem | Suburban | Suburban | 50°54'1.88"N | 3°50'8.84"E |
| Hillegem | Suburban | Suburban | 50°53'49.54"N | 3°51'2.78"E |
| Hillegem | Suburban | Rural | 50°53'56.02"N | 3°50'31.48"E |
| Atembeke | Rural | Urban | 50°46'27.80"N | 3°56'56.86"E |
| Atembeke | Rural | Urban | 50°46'34.36"N | 3°57'12.75"E |
| Atembeke | Rural | Suburban | 50°46'18.12"N | 3°56'45.69"E |
| Atembeke | Rural | Rural | 50°46'26.61"N | 3°57'25.91"E |
| Kalken | Rural | Urban | 51° 2'22.98"N | 3°55'17.30"E |
| Kalken | Rural | Urban | 51° 2'8.37"N | 3°55'28.65"E |
| Kalken | Rural | Suburban | 51° 1'58.32"N | 3°54'40.68"E |
| Kalken | Rural | Rural | 51° 2'11.30"N | 3°55'48.88"E |
| Kalken | Rural | Rural | 51° 2'0.41"N | 3°55'59.94"E |
| Melsen | Rural | Urban | 50°57'26.45"N | 3°42'10.19"E |
| Melsen | Rural | Urban | 50°57'21.87"N | 3°42'6.13"E |
| Melsen | Rural | Suburban | 50°57'5.73"N | 3°42'31.07"E |
| Melsen | Rural | Rural | 50°56'34.16"N | 3°43'21.73"E |
| Melsen | Rural | Rural | 50°56'48.99"N | 3°43'12.50"E |

Table S1: Overview of the 45 locations where lines were collected for the common garden experiment.

|  | Damage severity | | Proportion damaged leaves | |
| --- | --- | --- | --- | --- |
|  | GLMM: Multinomial | | GLMM: Binomial | |
|  | **F** | **P** | **F** | **P** |
| Plot | F_(2, 313)_= 0.22 | 0.7987 | F_(2, 17.43)_= 0.30 | 0.7419 |
| Subplot | F_(2, 315)_= 1.88 | 0.1543 | F_(2, 19.19)_= 0.67 | 0.5252 |
| Timing | F_(1, 317)_= 34.21 | **<0.0001** | F_(1, 313)_= 288.93 | **<0.0001** |
| Plot*Subplot | Did not converge | | F_(4, 14.3)_= 0.76 | 0.5699 |
| Plot*Timing | Did not converge | | F_(2, 307)_= 0.68 | 0.5072 |
| Subplot*Timing | Did not converge | | F_(2, 301)_= 0.54 | 0.5859 |
| Plot*Subplot*Timing | Did not converge | | Did not converge | |

Table S2: Results of the Type III-tests of Fixed Effects used for backwards model selection of (1) The GLMM (multinomial distribution, pconv=0.0001) testing the effect of plot, subplot and timing of herbivore exposure on severity of the suffered damage and (2) the GLMM (binomial distribution) testing the effect of the same factors on the percentage of leaves that were damaged.

|  | Dry aboveground biomass | |
| --- | --- | --- |
|  | LMM |  |
|  | **F** | **P** |
| Plot | F_(2, 17.4)_= 0.06 | 0.9454 |
| Subplot | F_(2, 20.5)_= 1.01 | 0.3829 |
| Treatment | F_(1, 257)_= 0.04 | 0.8430 |
| Plot*Subplot | F_(4, 16.8)_= 2.37 | 0.0938 |
| Plot*Treatment | F_(2, 256)_= 1.41 | 0.2463 |
| Subplot*Treatment | F_(2, 254)_= 0.61 | 0.5417 |
| Plot*Subplot*Treatment | F_(4, 250)_= 1.59 | 0.1777 |

Table S3: Results of the Type III-tests of Fixed Effects used for backwards model selection of the LMM testing the effect of plot, subplot and treatment on dry aboveground plant biomass.

|  | Seed weight per plant | | Seed heads per plant | | Seeds per seed head | |
| --- | --- | --- | --- | --- | --- | --- |
|  | LMM | | GLMM: Poisson | | LMM | |
|  | **F** | **P** | **F** | **P** | **F** | **P** |
| Plot | F_(2, 14.3)_= 4.08 | **0.0396** | F_(2, 18.49)_= 3.40 | 0.0554 | F_(2, 14.4)_= 2.06 | 0.1634 |
| Subplot | F_(2, 13.8)_= 0.94 | 0.4137 | F_(2, 18.62)_= 0.43 | 0.6547 | F_(2, 15.6)_= 0.58 | 0.5711 |
| Treatment | F_(1, 98.9)_= 3.19 | 0.0771 | F_(1, 112)_= 3.47 | 0.0653 | F_(1, 103)_= 0.06 | 0.8024 |
| Plot*Subplot | F_(4, 12.6)_= 0.63 | 0.6495 | F_(4, 15.45)_= 1.18 | 0.3582 | F_(4, 13.2)_= 0.32 | 0.8581 |
| Plot*Treatment | F_(2, 98.9)_= 6.38 | **0.0025** | F_(2, 112)_= 6.77 | **0.0017** | F_(2, 97.5)_= 0.13 | 0.8816 |
| Subplot*Treatment | F_(2, 97.6)_= 0.32 | 0.7239 | F_(2, 104)_= 0.66 | 0.5196 | F_(2, 102)_= 0.25 | 0.7769 |
| Plot*Subplot*Treatment | F_(3, 93.8)_= 0.41 | 0.7474 | F_(3, 101)_= 1.38 | 0.2534 | F_(3, 95)_= 0.29 | 0.8319 |

Table S4: Results of the Type III-tests of Fixed Effects used for backwards model selection of (1) the LMM testing the effect of plot, subplot and treatment on total seed weight per plant, (2) the GLMM testing the effect of the same factors on the total number of seed heads per plant, (3) the LMM testing the effect of the same factors on the average number of seeds per seed head per plant. Significant P values are printed in bold.

|  |  | Seed weight per plant | | | Seed heads per plant | | |
| --- | --- | --- | --- | --- | --- | --- | --- |
|  |  | LMM | | | GLMM: Poisson | | |
|  |  | **DF** | **t** | **Adj P** | **DF** | **t** | **Adj P** |
| A) | U-C x U-T | 95.9 | 3.49 | **0.0092** | 112 | 3.27 | **0.0175** |
|  | S-C x S-T | 101 | -1.48 | 0.6786 | 112 | -1.85 | 0.4358 |
|  | R-C x R-T | 99.9 | 0.88 | 0.9500 | 112 | 2.25 | 0.2222 |
| B) | U-C x S-C | 30.4 | -3.70 | **0.0046** | 33.03 | -3.45 | **0.0101** |
|  | U-C x R-C | 30.4 | -2.89 | 0.0516 | 27.53 | -1.95 | 0.3763 |
|  | S-C x R-C | 32 | -0.64 | 0.9875 | 42.29 | -1.38 | 0.7365 |
| C) | U-T x S-T | 20.3 | -0.41 | 0.9984 | 22.9 | -0.61 | 0.9905 |
|  | U-T x R-T | 19 | -1.39 | 0.7308 | 25.2 | -1.96 | 0.3735 |
|  | S-T x R-T | 18.5 | 1.08 | 0.8875 | 26.62 | 1.48 | 0.6757 |

Table S5: Tukey Post Hoc analysis of total seed weight and number of flowers per plant. U=Urban, S=Suburban, R=Rural, C=Control, T=Herbivore treatment. (A) Pairwise comparison of control and herbivore treatment within the same urbanisation level (B) Pairwise comparison of control groups across an urbanization gradient (C) Pairwise comparison of herbivore treatment groups across an urbanization gradient. Values in bold indicate significant differences (p≤0.05).
